# Torture-survivors’ experiences of healthcare services for pain: a qualitative study

**DOI:** 10.1177/2049463720952495

**Published:** 2020-09-09

**Authors:** Daniel Board, Susan Childs, Richard Boulton

**Affiliations:** 1 Chelsea and Westminster Hospital NHS Foundation Trust, London, UK; 2 Faculty of Health, Social Care and Education, St George’s, University of London and Kingston University, London, UK

**Keywords:** Pain, pain management, torture, healthcare experience, refugees

## Abstract

**Background:**

Increasing numbers of torture-survivors are presenting to UK healthcare services with persistent pain. However, there is a paucity of evidence surrounding the management of persistent pain among torture-survivors and their experience of healthcare services for pain is currently unknown. This qualitative study explores their experiences of services for managing pain, to inform clinical practice and service provision.

**Methods:**

Thirteen participants were recruited from a specialist pain clinic for torture-survivors in the United Kingdom. Utilising an ethnographic approach, data were collected via clinic appointment observations, interviews and medical records and analysed using inductive thematic analysis.

**Results:**

Three themes emerged in relation to torture-survivors’ experiences of healthcare services for pain: the *patient–clinician relationship*; *multiplicity of diagnoses and treatments*; *lack of service integration*. Participants described limited engagement in decision-making processes regarding their care. Lack of recognition of torture experience when diagnosing and treating pain, alongside multiple unsuccessful treatments, led to confusion, frustration and hopelessness. These issues were exacerbated by the disconnect between physical and mental health services.

**Conclusion:**

This study provides new insight into the challenges faced by torture-survivors when accessing healthcare services for pain. Our findings suggest current service provision is not meeting their complex needs. Clinical implications include the need for integrated care systems and better recognition of the influence of torture experience on persistent pain. Strategies to engage and empower torture-survivors in the management of their pain are suggested.

## Introduction

Despite being outlawed internationally, torture continues to occur in over 140 countries worldwide, taking place in both war-torn countries and in those without conflict.^1^ The United Nations defines torture as

any act by which severe pain or suffering, whether physical or mental, is intentionally inflicted on a person[. . .], when such pain or suffering is inflicted by or at the instigation of or with the consent or acquiescence of a public official or other person acting in an official capacity. . . (United Nations,^2^ p. 113)

Accurately documenting the true scale of torture and number of survivors is impossible. However, among refugees and asylum-seekers living in high-income countries such as the United Kingdom, estimates suggest between 27% and 44% have experienced torture.^3,4^

Many do not survive torture, but for those who do, the physical, psychological and social consequences are enduring. Physical consequences include restricted function and disability, with persistent (or chronic) pain present in over 80% of torture-survivors.^5,6^ Psychological sequelae include symptoms associated with anxiety, depression and post-traumatic stress disorder (PTSD), for which rates of depression and PTSD exceed 30%.^7^ These difficulties are compounded by the wider social circumstances of torture-survivors following displacement and forced migration. Torture-survivors residing in the United Kingdom, alongside other refugees and asylum-seekers, experience poverty, discrimination, fear of deportation, isolation, language barriers, and uncertainty of basic needs such as employment and housing.^8–10^

The healthcare needs of torture-survivors are understandably varied and complex.^11^ However, numerous obstacles to providing effective healthcare exist. Missed or unproductive appointments may occur due to poor literacy, language, memory or cognitive difficulties.^9,11^ The various social, legal and welfare issues torture-survivors face also regularly take precedence over seeking assistance with their health-related issues.^12^ Access to healthcare is also hindered by complex entitlement rules for refugees and asylum-seekers.^11^ Individuals are often reluctant to seek healthcare support, fearing excessive charges for services, or that enquiry into their eligibility to access healthcare may negatively influence asylum-seeking processes and even lead to deportation.^13–15^

Importantly, when torture-survivors do engage with healthcare services, there is often a failure to identify them as such. Many are reluctant to disclose their experience of torture due to shame, fear of judgement or disbelief, avoidance of re-experiencing trauma during disclosure, or through a lack of knowledge regarding how trauma can impact health.^16,13^ Clinicians may also be reluctant to enquire regarding a history of torture, possibly due to feeling practically or emotionally unprepared to explore and deal with the associated issues.^17^ When torture experience remains undisclosed, addressing the healthcare needs of torture-survivors becomes more difficult. It is suggested this may lead to negative outcomes for patients,^18^ although this has not been explored empirically.

Among torture-survivors who do access healthcare services, the prevalence of pain-related issues is high.^5,6^ Torture-survivors commonly report persistent pain throughout the musculoskeletal system. It can be focal or widespread, with the most common pain sites including the head, spine and limbs.^6,18,19^ Reports of abdominal, pelvic and genitourinary pain are also common.^18^ Despite the high prevalence of pain, the psychological consequences of torture often take precedence and receive considerable attention within refugee healthcare, with pain under-recognised in both clinical and research settings.^12,20^ When it is recognised, a traditionally poor understanding of pain often leads it to be attributed to psychological distress, an outdated and unfounded view, given current knowledge of pain mechanisms.^18^

Rehabilitation after torture is a human right.^2^ However, outcomes from treatment of pain are typically poor and there is currently no good evidence to support or refute any intervention for managing pain.^12^ Much of the research that is available has been descriptive,^18^ with the few experimental studies underpowered and lacking scientific rigour.^12^ Systematic exploration of the perspectives of torture-survivors themselves is also scarce and sheds little light on their experience of healthcare.^21,22^ This is an important gap in the literature, as it is only through interpreting their experience that we may gain a truly detailed understanding of how and why current service provision is not meeting their needs.

The present study’s aim was therefore to gain a deeper understanding of torture-survivors’ experiences of services for managing pain, to inform clinical practice.

## Methods

### Design

This qualitative study utilised an ethnographic approach, amalgamating data from non-participant observations of clinical appointments, in-depth semi-structured interviews and medical record data. Thirteen torture-survivors with persistent musculoskeletal pain took part in the study, recruited from a specialist pain clinic in the United Kingdom.

The study was designed with feedback from expert clinicians, researchers and charity organisations with firsthand experience of the issues torture-survivors face.

### Position of the researcher

The study was undertaken as part of an academic award (Master of Research in Clinical Practice) sought by the Principal Investigator (D.B.). D.B. is a White British, male physiotherapist, who had prior experience working with torture-survivors at the study site. It is acknowledged that pre-existing assumptions regarding pain, healthcare and awareness of the wider issues facing torture-survivors had the potential to influence the study findings.^23^ Strategies to mitigate this included reflexive accounts of the researcher, gathering of data from multiple sources, cross-checking of transcripts amongst the research team, and a constant dialogue with clinical and academic peers to allow for scrutiny of the project and its findings.^23^ D.B. received extensive training in qualitative research methods via the academic award. The Chief Investigator, (R.B.) a sociologist and experienced qualitative researcher, provided additional training and supervision to D.B. during data collection and analysis.

### Setting and participants

The study setting was a specialist multidisciplinary pain clinic for torture-survivors, set within the wider pain management service of a metropolitan hospital in the United Kingdom. Patients accessing the service were predominantly men of working age from Middle Eastern and South Asian countries including Iraq, Iran, Syria and Afghanistan. Fewer but still significant numbers originated from North and East Africa and Eastern Europe.

A purposive sampling strategy was used. Eligible patients were older than 18 years, had musculoskeletal pain related to torture experience for a duration of over 6 months, identified as a survivor of torture and spoke English or Arabic. Patients were screened and recruited by a clinical psychologist at the clinic, before being introduced to the researcher (D.B.). If during screening it was felt participation might be detrimental to patients’ psychological wellbeing, they were not approached. This decision relied on the clinical judgement of the psychologist upon meeting the patient, with consideration of any documented psychological history and self-reported measures of mood and anxiety routinely completed at the clinic. All eligible patients attending the clinic between May and July 2018 were invited to participate.

Detailed participant information was provided in participants’ primary language (English or Arabic). Where required, professional interpreters were present for all face-to-face contact.

### Ethical considerations

Given the vulnerability of the study population, a number of safeguards were in place to ensure participation was not detrimental to participants. This included access to a clinical psychologist, regular monitoring for psychological distress and debriefing upon completion of participant involvement in the study. Written informed consent was obtained from all participants. The study received favourable opinion by the National Health Service (NHS) London Brent Research Ethics Committee (Reference: 18/LO/0420).

### Data collection

All data were collected on site by D.B. To provide flexibility, participants could opt into any combination of three data collection methods: clinic appointment observations, in-depth semi-structured interviews and medical record access.

Non-participant observations were conducted during participants’ initial or follow-up appointment at the clinic, providing the researcher with firsthand experience of the setting and interactions between participants and clinicians. Recorded information included participant verbal and non-verbal communication, behaviours, direct quotes, a diagram of the setting and reflexive accounts of the researcher. Field notes were recorded using an observation data collection tool (Supplemental Appendix A).

In-depth semi-structured interviews were conducted at the clinic at a time that suited participants. Interviews were audio-recorded and guided by an interview schedule. The interview schedule was informed by a systematic literature review completed during protocol development, and refined following feedback from researchers, clinicians and charities working directly with torture-survivors. Open-ended questions allowed for in-depth exploration of participant experiences, perceptions and understanding of more complex processes such as motivation of behaviours noted during observation.^24^ Interviews started with general questions regarding pain and its impact (e.g. Can you tell me about your pain? How does it affect you?), before moving on to experiences of healthcare services (e.g. Who do you go to when you’re in pain? What treatments have you tried? What was your experience of these services?). Professional interpreters were utilised where required and briefed beforehand regarding the study aim and their role.

Relevant data from participants’ medical records were also collected to provide complementary information that could not be gathered from interview or observation.^24^ This included clinic referral letters, clinic outcome letters and routinely completed self-report questionnaires regarding anxiety and depression, severity of pain and impact on function.

Interview transcripts, observation field notes and medical record data were collated and reviewed by the research team throughout data collection. Recruitment was halted once it was felt that the data set was sufficiently rich to address the research aim.

### Data analysis

Following data collection, interview transcripts were analysed alongside observation field notes using NVivo data analysis software.^25^ To allow findings to emerge from the data, analysis followed the six stages of inductive thematic analysis as guided by Braun and Clarke.^26^

Interview transcripts and observation field notes were read repeatedly to provide familiarity, before a list of codes were generated from the data. Transcripts were read and coded independently by D.B. into themes and sub-themes, through line-by-line coding with regular discussion and revision with R.B. Themes and sub-themes were then compared across participant data sets and the entire data set, leading to generation of an initial thematic map containing all relevant themes with supporting codes. Themes were reviewed and refined before final defining and naming took place. This involved the development of a final thematic map constructing a coherent and consistent account of the final themes.

Medical record data were used to inform interview questioning and provide supplementary information during presentation of the study findings.

Data analysis was led by D.B. Cross-checking of transcripts and a constant dialogue between all authors took place to ensure findings were representative of the data.

## Results

Following screening, 19 patients were deemed eligible and invited to participate, of which 14 took part in one or more method of data collection. One participant withdrew their consent after observation but prior to interview. They were not engaged further, and all previously collected data were destroyed. This resulted in a final sample of 13 participants. Individual characteristics are described in Table 1.

**Table 1.** Participant characteristics.

Participant	Gender	Region of origin and interpreter required (Y/N)	Age (decade)	Pain duration (years)	Pain location(s)^a^	Data collected (MR, Obs, Int)
PT1	Male	Middle East (Y)	40–49	20	Lower back and both knees	MR - Obs - Int
PT2	Female	Middle East (N)	40–49	3	Widespread	MR - Obs - Int
PT3	Male	Southeastern Europe (N)	50–59	10	Widespread and migraines	MR - Int
PT4	Male	North Africa (N)	50–59	18	Widespread	MR - Obs - Int
PT5	Male	Middle East (Y)	60–69	25	Widespread	MR - Obs - Int
PT6	Male	Middle East (N)	40–49	11	Lower back, neck and shoulders	MR - Obs - Int
PT7	Male	Middle East (Y)	30–39	8	Neck and both shoulders	MR - Obs
PT8	Male	Middle East (Y)	30–39	12	Widespread	MR - Obs - Int
PT9	Male	Middle East (Y)	40–49	17	Widespread	MR - Obs
PT10	Male	Middle East (Y)	30–39	14	Widespread	MR - Obs
PT11	Male	Middle East (Y)	50–59	18	Widespread	Obs
PT12	Female	South Asia (N)	20–29	5	Lower back, wrists and ankles	MR - Obs - Int
PT13	Male	Middle East (N)	40–49	20	Widespread	MR - Obs - Int

MR = medical records, Obs = clinic observation, Int = interview.

a Pain location(s) – five or more body areas and/or pain moves around the body was considered widespread.

Data collected included 12 clinic observations lasting 25–90 minutes (average 56 minutes), 9 interviews lasting 13–55 minutes (average 38 minutes) and access to 12 medical records.

**Figure 1.**
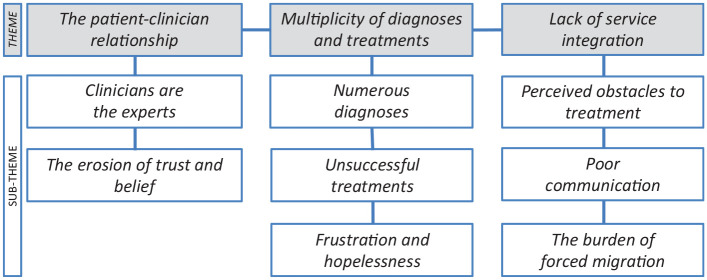
Themes and sub-themes.

Three main interlinking themes emerged in relation to torture-survivors’ experiences of services for managing pain: *the patient–clinician relationship; multiplicity of diagnoses and treatments; lack of service integration*. Themes and sub-themes are depicted in Figure 1.

Themes are presented sequentially below to build a picture of torture-survivors’ experiences of the healthcare system, from individual relationships to the influence of organisational processes.

### The patient–clinician relationship

Participants described clinical encounters with little or no active involvement in decision-making processes regarding their care. This appeared to be driven by participants’ respect for medical authority and trust in clinician expertise. For many, this trust and belief in clinicians slowly eroded over time following numerous differing diagnoses and unsuccessful treatments.

#### Clinicians are the experts

Nearly all participants described perceiving clinicians as ‘professionals’ (PT3) or ‘experts’ (PT1) and, as such, felt they should adhere to whatever treatments were suggested.

PT6 Interview ‘The professional people should tell me what to do’

PT13 Observation ‘I am totally in your hands. Wherever you direct me I will follow’

There was subsequently limited evidence of previous collaborative or patient-led decisions regarding treatment. Participants instead described experiences whereby treatments were prescribed by clinicians and adhered to diligently. Some even reported a sense of guilt if they wavered from prescribed treatments.

PT4 Interview ‘I don’t want to tell my GP, but when I feel good I don’t take my medications’

PT1 interview ‘They say do this, do this, do this. I take this tablet and do this cream. I say no problem, because you are doctor. I’m not doctor. I have to listen to the doctor’

#### The erosion of trust and belief

Despite most participants describing high levels of trust in clinicians, participants also discussed how repeated encounters with no improvements in their pain led to disappointment and reduced trust or belief that medical professionals have the answer.

PT6 Interview ‘Maybe I trust some of them, but to be honest it’s a regular thing when I going to my GP, it’s disappointing you know. Why is I going? Just give me medicine and the pain is still coming, but why?’

PT1 Interview ‘I getting tired, exhausted, going in and out. Getting stressed. You don’t know which trying to help you and which not’

These findings highlight the impact that perceptions of medical authority and clinician expertise had within the patient–clinician relationship, influencing adherence to treatment and a lack of engagement of torture-survivors in decision-making processes. Repeated negative experiences led to a reduction in trust and belief in clinicians. These negative experiences are explored further in the following themes and include receiving varying and often conflicting diagnoses, repeated unsuccessful treatments and difficulties accessing and engaging with services.

### Multiplicity of diagnoses and treatments

All participants described encounters with multiple healthcare professionals regarding their pain, often in uni-disciplinary settings. These included visits to general practitioners and pharmacists, alongside referrals to physiotherapy, trauma and orthopaedics, rheumatology, cardiology and multidisciplinary pain management services. All participants had a current diagnosis of depression, generalised anxiety disorder or symptoms of PTSD, and had undergone or were receiving treatment for psychological comorbidities. Participants described receiving varying diagnoses and experiencing numerous unsuccessful treatments, often leaving them confused regarding the cause of their pain and frustrated at the lack of any improvement.

#### Numerous diagnoses

Participants described receiving varying diagnoses, with all given at least one diagnosis involving a structural issue and a third being advised their pain was due to psychological issues.

PT13 Interview ‘One of them said you have Fibromyalgia, other one said you have a slipped disc, another one said you have PTSD. Everyone has given me different type of diagnosis but no-one’s exactly tried to help me’

PT2 Observation ‘They said it’s the stress affecting my body and slipped discs’

These descriptions were supported by medical record data, with participants’ clinic referrals implicating predominantly structural issues as the cause of pain.

PT5 Referral letter ‘Chronic low back pain due to degenerative changes in the spine’

PT4 Referral letter ‘Lumbar disc derangement’

When psychological factors were considered conducive to pain experience, it was often done so separately, as if a separate issue, or included in the ambiguous term ‘Biopsychosocial overlay’.

PT2 Referral letter ‘Impression: 1. widespread neuropathic pain [. . .] 2. Biopsychosocial overlay’

PT9 Referral letter ‘Impression: 1. Chronic centralizing Left unilateral body pain [. . .] Query marked biopsychosocial overlay’

The varying and often conflicting diagnoses left participants confused about the cause of their pain, with some describing feeling disbelieved they were in pain due to the lack of an obvious identifiable cause.

PT7 Observation ‘Every time I go to hospital they say nothing wrong with you, go home. What should I do?’

PT1 Interview ‘They said no problem for both knee and back. I said no, look, I have pain, too much pain’

#### Unsuccessful treatments

Participants described experiencing numerous interventions, including medications, injections and exercises, with most providing limited or no benefit and some coming with unwanted side-effects.

PT3 Interview ‘With the surgery and all those injections I had [. . .] I had so many of those and they were so painful, the steroid injections, and they didn’t really help anything’

#### Frustration and hopelessness

The numerous diagnoses and unsuccessful treatments left many participants disheartened and sceptical of achieving positive future outcomes. PT13 described receiving referrals to various services and being provided with only short-term solutions to his pain, as a result describing feelings of frustration and hopelessness.

PT13 Interview ‘At the end of the day, if I still had the same pain after 10 years, you know, it’s just giving a lollypop to a crying baby. That’s it. But when that lollypop finished day after, when that baby start crying again, what are you gonna do? You cannot give lollypop every day [. . .] so people always trying to give me lollypops and I had enough’

These findings highlight the range of healthcare professions participants had encountered regarding their pain, often receiving conflicting diagnoses and experiencing numerous unsuccessful treatments. In many cases this left participants confused regarding the cause of their pain, frustrated at the lack of improvement and hopeless regarding positive future outcomes.

### Lack of service integration

With all participants having experienced a range of healthcare services for both pain and psychological comorbidities, evidence of a disconnect between various physical and mental health services became apparent. Participants described how both pain and psychological comorbidities were frequently experienced as obstacles to engaging with healthcare services. Poor communication between services also influenced participants’ ability to access and benefit from them. These issues were further impacted by difficulties experienced in relation to participants’ refugee and asylum status.

#### Perceived obstacles to treatment

When discussing experiences of healthcare, participants described how both pain and low mood affected their ability to engage with services.

PT2 Interview ‘I did try (physiotherapy), but I am in pain’

PT6 Interview ‘When I don’t have good mood I don’t like to go (to hospital)’

Psychological issues such as severe depression or suicidal ideation were frequently viewed by physical health services as a barrier to engaging with their services. Participant descriptions supported this, with one participant explaining how being deemed too psychologically unwell to access a group pain management programme left him feeling distressed.

PT4 Interview ‘I was supposed to be with the group, but I wasn’t qualified to be with the group and it was another pain for me. Really it was another pain’

#### Poor communication

It was also noted that poor communication between services was impacting participants’ ability to access and engage with them. During PT10’s clinic observation, clinicians had requested an onward referral. However, having not been actioned, clinicians were unable to offer further advice until this had taken place. In response, PT10 reported ‘. . . it is really stressful for me’.

The presence of these communication issues was supported by medical record data. The excerpt from PT2’s referral letter shows how she waited an extra 5 months for an appointment due to a lost referral. During PT7’s appointment, clinicians were frustrated at the lack of information included in the referral, making it difficult to identify potentially beneficial changes to his medications.

PT2 Referral letter ‘Our colleagues at *** referred this patient to your pain clinic in December of last year. Unfortunately this referral seems not to have got through [. . .]’

PT7 Clinic letter ‘Unfortunately, when the referral was made from ***, they did not send your referral letters to us. This is quite unfortunate as the consultation was significantly hampered by lack of information’

#### The burden of forced migration

Participants described how difficulties accessing and engaging with healthcare services combined with issues relating to refugee and asylum status. PT8 described a complicated process when accessing services on arrival in the United Kingdom, while PT12 described how asylum-related concerns exacerbated her psychological distress, in turn leading her to experience physical pain.

PT8 Interview ‘In the hotel there was a doctor. He told me, [. . .] I cannot for example give you any medication to treat the pain, because first we have to make a diagnosis and that should be done by another doctor. [. . .] Then when I registered with GP he gave me Co-codamol, then after that when I move to ***, my GP here change it to Tramadol. [. . .]My GP refer me to Physiotherapist. [. . .] But when I start to do exercise I become breathless and sweat. She told me that if this the case you have to take rest, then ask your GP to refer you back to us’

PT12 Interview ‘Worrying about what’s going on with my visa [. . .] that’s the initial thing. So after that it’s all coming up like a chain . . . what happened in the past, it’s all coming back [. . .] When I’m reliving it I get the pain’

These findings highlight the complex, interactive relationship between participants’ pain, psychological distress and wider social difficulties, all impacting their ability to access and engage effectively with healthcare services. These issues were compounded by poor communication between various services, demonstrating a lack of service integration.

## Discussion

The torture-survivors studied experienced a variety of challenges when accessing and engaging with healthcare services for pain, describing mostly negative experiences. Participants described clinical encounters with little or no engagement in decision-making processes regarding their care. Their initial trust and belief in clinicians, and healthcare in general, eroded over time due to the multiplicity of often conflicting diagnoses and unsuccessful treatments. A number of institutional shortcomings were identified through observation and medical record data, with participant descriptions of numerous referrals, diagnoses and unsuccessful or delayed treatments, confirming the impact these shortcomings had on them. Ultimately, this left many frustrated and hopeless at achieving any meaningful improvements in their pain.

The psychological impact of torture is an important consideration when exploring the patient–clinician relationship. Many torture-survivors experience long-term psychological distress including symptoms of depression, anxiety and PTSD,^7^ related not only to the inciting torture but also to the various social, legal and welfare issues they face.^9,10^ Many also report difficulties in expressing themselves and hold a generalised mistrust of others, especially those in positions of authority.^13^ It is understandable that such difficulties might translate into clinical interactions, potentially hindering effective communication between torture-survivors and clinicians.^16^

Beyond the psychological barriers, cultural beliefs regarding health and healthcare may also impede communication and shared decision-making in intercultural patient–clinician relationships.^27–29^ For some ethnic minority groups, cultural norms exist requiring deference to healthcare professionals’ authority.^16,30^ The need to show respect for, and avoid conflict with clinicians, has been shown to facilitate passivity and reduce participation in clinical encounters,^30^ even when patients wish to be recognised as experts of their illness.^31^ This is consistent with participant descriptions in the present study and supported by researcher reflections regarding participants’ behaviour. During interviews, participants were keen to express their views regarding treatment. Many questioned prescribed treatments and enquired regarding interventions they thought might be beneficial. This was not noted during clinic observations, suggesting participants were more comfortable putting forward their own views when not in the presence of the treating clinician.

When discussing culture and ethnicity, it is also important to highlight the role of implicit bias in shaping patient–clinician interactions. Implicit bias occurs when unconscious stereotypes and prejudices influence behaviour, usually negatively, to disadvantage a person or group based on a common trait or characteristic.^32^ While explicit bias is generally becoming less prevalent within healthcare, implicit bias is still common, even among clinicians who outwardly deplore such prejudicial views.^33^ Implicit bias is shown to influence clinical interactions and decision-making processes,^33^ contributing to the inequitable healthcare experienced by ethnic minority groups.^34^ Minority patients, particularly those with limited English, are less likely to engender empathic responses from clinicians.^27^ Moreover, clinicians have been shown to be less positive, provide less patient-centred care and are less likely to encourage patient participation during interracial encounters, when compared with same-race interactions.^33,35^ Torture-survivors are particularly vulnerable to such biases due to the widespread negative perception of refugees and asylum-seekers in the United Kingdom, ^36^ often portrayed as a threat to the stability of community and sovereignty and a burden on the healthcare system.^36,37^ Despite their complex health needs,^11^ these views invert concerns such that the host population is perceived as ‘at risk’, as opposed to the vulnerable displaced.^37^ Furthermore, patients’ responses to such bias, alongside their own biases, might compound ineffective patient–clinician interactions, leading to reduced healthcare-seeking behaviour and ultimately poorer health.^33^

Determining the exact mechanisms influencing patient–clinician interactions and decision-making is especially difficult, given the varying and nuanced factors involved. Therefore, in light of the current shortcomings in managing torture-related pain, the relationship between patient and clinician is a cornerstone of service provision that warrants further investigation.

Participants described receiving a multitude of diagnoses and treatments for their pain, utilising mostly uni-disciplinary biomedical approaches. The development of persistent pain is proposed to be the result of various neurobiological, psychological, environmental and social factors.^38–40^ The pathophysiology of torture-related pain is not fully understood. However, the infliction of intense and prolonged physical and psychological distress during torture is considered an important risk factor.^18^ When combined with the lasting psychological impact of torture, and the fact many displaced torture-survivors are then deprived of protective factors including social support and access to healthcare, the high prevalence of persistent pain is perhaps unsurprising.^18^ Despite this, participants in the present study were assigned predominantly biomedical diagnoses as the cause of their pain, with poor consideration of torture experience and its wider psychological and social consequences.

Although receiving a diagnosis has been highlighted as positive through legitimising patients’ suffering,^41^ many persistent pain conditions do not have underlying pathoanatomical causes that adequately explain a pain experience.^42,43^ Many structural abnormalities proposed to be the cause of pain in this study, including degenerative changes and disc bulges, are commonly seen in pain-free populations.^44^ Thus, assigning pain from torture to be the result of pathoanatomical change ignores the pivotal role of torture experience and its multifaceted physical, psychological and social consequences. Furthermore, labelling the cause of pain with words such as ‘degenerative’ or ‘derangement’ may serve to increase fear, instilling beliefs the body is in some way damaged, fragile or needs protecting.^45^ This is important in a population where such catastrophic beliefs are posited to contribute to the maintenance of comorbid pain and PTSD.^46,47^

It was not clear what drove the overly biomedical approach to pain diagnosis and treatment, a question perhaps better explored through the clinician’s lens. It is possible that barriers to effective communication, as discussed above, could lead to superficial exploration of torture-survivors’ pain and suffering. However, an important consideration is the potential failure to identify a history of torture at all. There is currently no reliable data regarding the number of torture-survivors accessing healthcare services in the United Kingdom, with only a few small studies conducted elsewhere.^48–50^ While these cannot be generalised to UK healthcare, those cited found the prevalence of torture among foreign-born people in healthcare settings to be between 6% and 11%. Perhaps more importantly, a majority had never disclosed their torture experience, nor had clinicians asked about a history of torture. If torture experience remains undisclosed, the wider psychological and social factors influencing torture-survivors’ pain are likely to remain unaddressed. This could explain the oversimplified biomedical approach to pain observed, supporting the argument that failure to identify torture during clinical encounters contributes to negative outcomes for patients.^18^

A lack of integration of healthcare services is not a new finding, nor is it exclusively experienced by torture-survivors. Indeed, addressing such shortcomings and delivering better person-centred and co-ordinated care has been a priority for the UK NHS for many years.^51–53^ However, our findings would suggest current care provision for torture-survivors with persistent pain is not yet meeting these standards. Torture-survivors are likely to suffer from a range of interconnected physical and mental health difficulties, alongside human rights issues including poverty, racial discrimination and asylum-related challenges.^18^ Pain should therefore be viewed within the wider context of these issues, as failing to do so may undermine treatments for managing pain and contribute to negative outcomes for patients.

This study provides insight into an important area of healthcare not previously explored, providing a detailed first-person perspective of torture-survivors’ experiences of services for managing pain. It is important to note that other complex and vulnerable populations may face similar experiences and it may be difficult to distinguish many of these from those faced by torture-survivors. However, it is precisely this ambiguity that requires further attention, in order that we might fully understand the extent of pain-related issues resulting from torture.

Torture-survivors show incredible resilience in the face of great adversity. Nonetheless, instigating meaningful change in the care of torture-related pain is reliant upon action by clinicians and service-providers. We therefore suggest a number of recommendations. First, service-providers should strive to deliver better integrated care, where physical and mental health support can be provided simultaneously, alongside social support. Second, clinicians should be aware of the possibility their patients may have experienced torture, in an effort to aid disclosure and facilitate appropriate management. Alongside a thorough subjective assessment, a key consideration in identifying torture is having an awareness of the likelihood of torture being carried out in the patient’s country of origin,^8^ information easily accessible online from organisations including Freedom from Torture and Amnesty International.^54,55^ This should then be followed by gentle enquiry. Questions such as ‘Can you tell me why you came to the UK?’, ‘Were you ever treated badly in your home country?’, or ‘Have you ever been arrested or put in prison?’ can assist in opening a dialogue regarding possible torture experience. People who have not been tortured are unlikely to mind such questioning, while those who have are often willing to disclose to a clinician who is open and caring enough to ask.^13^ Finally, clinicians should reflect upon the potential for unconscious bias to influence their practice, while employing bias-reducing strategies such as deliberate perspective-taking and individuation.^56^

The study has a number of limitations. Given the nature of the population and limited throughput of patients at the study site, the sample studied was relatively small. However, this was considered during study design, with multiple sources of rich data serving to strengthen the credibility of the findings.^23^ Furthermore, the sample predominantly included those with limited positive responses to previous treatments. This may lead to differing experiences of those living well with pain, or who have responded positively to pain interventions. Future research should therefore consider the experiences of torture-survivors within a variety of settings, including primary care and non-clinical environments. Clearly defined eligibility criteria were used to identify participants. However, recruitment via a healthcare professional, into a study examining healthcare experiences, carries the risk that patients might be selected on the basis of their amenability, or their likelihood of holding positive views of healthcare. Every effort was made to mitigate this through prior briefing of the clinician regarding the recruitment process. Nonetheless, this could influence the trustworthiness of our findings. The use of interpreters during data collection comes with its own limitations. For example, the conceptual meaning of participants’ responses can be lost or diluted through literal translation.^57^ Although unavoidable, it was ensured all interpreters had appropriate qualifications, were briefed on the study aims and variations in regional dialect were accounted for where possible.

## Conclusion

Torture-survivors face a variety of challenges when accessing and engaging with healthcare services for pain. The findings demonstrate a lack of engagement of torture-survivors in decision-making processes regarding their care. Poor recognition of torture experience when diagnosing and treating pain may contribute to an oversimplified biomedical approach to pain management, in turn leading to negative outcomes for patients. These issues are exacerbated by the disconnect between physical and mental health services, leaving torture-survivors struggling with pain currently occupying a precarious position within our compartmentalised healthcare system.

## Supplemental Material

Supplemental_material – Supplemental material for Torture-survivors’ experiences of healthcare services for pain: a qualitative study Click here for additional data file. Supplemental material, Supplemental_material for Torture-survivors’ experiences of healthcare services for pain: a qualitative study by Daniel Board, Susan Childs and Richard Boulton in British Journal of Pain
