# Supplementary material for: Torture-survivors’ experiences of healthcare services for pain: a qualitative study
Source: Br J Pain. 2020 Sep 9;15(3):291–301. doi: 10.1177/2049463720952495 (PMC8339948; doi:10.1177/2049463720952495)
Supplement: Supplemental_material – Supplemental material for Torture-survivors’ experiences of healthcare services for pain: a qualitative study [file Supplemental_material.pdf]

Appendix A. EPSOT Observation Data Collection Tool

(Modified from Creswell and Poth, 2017 – pg 171)

Introduction: Re-introduce researcher and role/ aims of observation. Highlight that observation can be discontinued at any time. Questions welcome at the end.

KEY:

Doctor	D	Nurse	N
Psychologist	P	Physiotherapist	PH
Patient	PT	Interpreter	I
Other:			

**Participants**<br>Patient (PIN):<br>Clinicians:<br>Interpreter:	Date:<br>Time:<br>Length of Observation:
For details of the room layout see sketch overleaf	
Pre-observation reflections:<br>*(expectations/ bias, expected role – observer/participant, relationship with participant(s))*	
Descriptive Notes	Reflective Notes
Post-observation reflections:<br>*(role as observer/ participant, themes, what did you learn, what more would you like to know)*	

Thank participants, offer time for questions to researcher.

Questions:

Room Layout:
